# Single-Stroke Attachment of Sheets to Tube Ends Made from Dissimilar Materials

**DOI:** 10.3390/ma14040815

**Published:** 2021-02-08

**Authors:** Luis M. Alves, Tiago J. Reis, Rafael M. Afonso, Paulo A.F. Martins

**Affiliations:** IDMEC, Departamento de Engenharia Mecânica, Instituto Superior Técnico, Universidade de Lisboa, Av. Rovisco Pais, 1049-001 Lisboa, Portugal; luisalves@tecnico.ulisboa.pt (L.M.A.); Tiago.h.reis@tecnico.ulisboa.pt (T.J.R.); rafael.afonso@tecnico.ulisboa.pt (R.M.A.)

**Keywords:** joining by forming, tube-to-sheet joints, finite elements, experimentation

## Abstract

This paper presents a new joining method by a forming process for attaching sheets to tube ends. The process consists of two different forming stages carried out sequentially in a single stroke. Firstly, the free tube end is flared by compression with a contoured die, then is squeezed (indented) against the sheet surface to create a mechanical interlocking. The new process is carried out at an ambient temperature and, in contrast to existing joining by forming operations based on tube expansion, it avoids seal welds, tube protrusions above the sheet surfaces, and machining of grooves on the sheet holes to obtain the form-fit joints. The paper starts by analyzing the process deformation mechanics and its main operating variables and finishes by presenting examples that demonstrate its effectiveness for attaching sheets to tube ends made from polyvinylchloride and aluminum. Experimental and numerical simulation work provides support to the presentation.

## 1. Introduction

**Figure 1:**
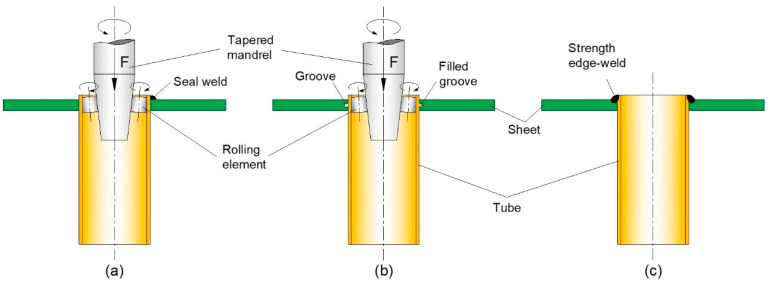
Commonly used processes for attaching metal sheets to metal tube ends: (**a**) Expansion with a roller without (left) and with (right) seal welding; (**b**) Expansion with a roller using sheet holes with machined grooves; (**c**) Strength fusion welding.

The processes for attaching metal sheets to metal tube ends can be classified into two main groups: (i) joining by forming processes and (ii) fusion welding processes. Joining by forming processes is based on the expansion of the tubes using hydraulic, mechanical, explosion or magnetic pressure [1,2,3,4,5]. Mechanical expansion with a roller [4] is the most widely used process and the resulting sheet to tube end attachments are built upon force-fit (also known as ‘interference-fit’) joining mechanisms (Figure 1a).

Force-fit joining mechanisms prevent detachment by relative movement of the metal tubes and sheets through frictional forces originated from the residual normal pressures that are created at the contact interfaces after unloading. In some applications requiring leak tightness, force-fit joining may be combined with seal welding of the tube ends (Figure 1a) [6]. Seal welds can be done before or after tube expansion. If they are made before, they may be damaged during expansion and fail in service by fatigue cracking [7], but if they are made after, the tubes may get loosened due to changes in dimensions caused by the heating–cooling cycle of welding.

In other applications requiring both leak tightness and pull-out resistance, grooves can be machined in the sheet holes to combine force-fit with mechanical interlocking (also known as ‘form-fit’) joining mechanisms (Figure 1b) [4].

The utilization of fusion welding processes for attaching metal sheets to metal tube ends is usually carried out by means of full-strength welds produced by gas tungsten arc welding (GTAW) (Figure 1c). The strength of the fusion-based joints is greater than the axial tube strength [6] but their use is limited to tubes and sheets made from similar metals to avoid weldability problems due to the incidence of hard and brittle intermetallic compounds. Fusion welding also requires the use of clamps and jigs to prevent distortions induced by the heating–cooling cycles.

Table 1 summarizes the main characteristics and features of the two main groups of processes that are commonly used for attaching metal sheets to metal tube ends. As seen, the joining by forming group is subdivided into two different subgroups as a function of the type of joint: (i) force-fit and (ii) combined force and form-fit joints.

The main conclusions to be drawn from Table 1 are that (i) the available processes are only focused on metals and (ii) the main advantages associated with the utilization of force-fit joints are compromised by the need to use seal welds to ensure leak tightness and to enhance the pull-out resistance. The combination of force and form-fit joints avoids welding but compromises productivity and final cost due to the necessity of machining grooves in the sheet holes prior to expansion with a roller.

Current trends in the manufacturing of lightweight, high performance, multi material structures are stimulating new solutions that seek to extend the applicability domain of the joining processes of Table 1 to the connection of dissimilar materials with significant differences in strength. In case of sheet-tube components involving polymers and metals, the state-of-the-art review on the joining of dissimilar materials by Martinsen [8] allows concluding that joining by forming has potential to be an alternative to existing adhesive bonding processes. This is important because the use of adhesives is limited by surface preparation, by temperature and environmental conditions, by difficulties in disassembling the individual parts at the end of service life and by the use of clamps, jigs and fixtures to lock and hold the sheets and tubes in position during the curing time.

Under these circumstances, the overview on recently proposed joining by forming processes with potential for connecting sheets to tubes starts by the work of Lee et al. [9], who performed the connection of discs (or sheets) with beveled surface rings by compression against the outer surface of tubes with serrated teeth. The process was successfully applied to connect aluminum and steel parts but its applicability to polymers and metals is unlikely to be feasible due to lack of filling and to the risk of causing excessive deformation on the polymer and of loosening due to polymer springback. Moreover, the process requires previous forming or machining of the beveled rings and knurling of the tube to fabricate serrations.

Another solution worthy of consideration is the new method for forming flanges on hollows parts by radial extrusion proposed by Winiarski et al. [10]. The process was successfully applied to connect metal rings (sheets) to the metal tube ends, and is based on a force-fit joining mechanism relying on the residual normal pressures that are created at the contact interfaces after unloading. Application of this process to sheet-tube attachments made from dissimilar materials is feasible but there is a risk of cracking the outer sheet edges and an elevated risk of sheet buckling after removing the component from the tool. Leak tightness requirements may also require the need of additional operations.

The method for joining a sheet to a tube through end curling proposed by Agrawal and Narayanan [11] may also be considered. The process was developed for joining metal sheets and tubes and the working principle can be extended for applications involving polymers and metals. However, its extension is limited by the large protrusions of the curled tube above the sheet surface and by difficulties in the disassembly of the tools after process completion.

**Figure 2:**
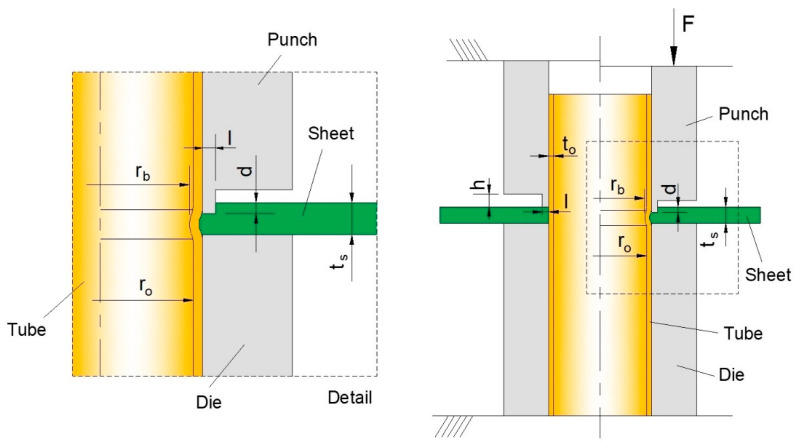
Schematic representation of the process and main variables for joining a sheet to a tube away from its end by annular sheet squeezing at the open and closed tool positions [12,14].

In fact, the challenge to be addressed in this paper is to propose a joining by forming process for attaching sheets to tube ends made from dissimilar materials (polymer and metals) in a single operation by means of a form-fit (mechanical interlocking) predominantly based mechanism. The process should also prevent the formation of material protrusions above the sheet surface and the need for additional seal welding operations. As a result of this, the source of inspiration for the process to be presented in this paper was the work previously done by the authors in attaching metal sheets to metal tubes, away from the tube ends [12,13] (Figure 2) and its subsequent extension to dissimilar materials [14,15].

The development of the joining by forming process shown in Figure 2, allowed authors to conclude that a deformation zone parameter $\Delta \;=\;t_{s}/l$ defined as the ratio of the sheet thickness $t_{s}$ to the cross-section recess length l of the punch can be utilized to characterize plastic material flow inside the sheet thickness, and to establish the fundamental design guidelines to produce sound form-fit joints. Results showed that values of Δ = 2–3 corresponding to $t_{s}\;=\;5$ mm and l = 1.7–2.5 mm and entailing a combination of inhomogeneous and homogeneous plastic material flow could produce near symmetric sheet-tube contact interfaces with a good amount of material squeezed and sufficient constriction from the external undeformed regions of the sheets to prevent material from bending [12,13].

The above-mentioned values of Δ were subsequently found adequate for attachments involving sheets and tubes made from dissimilar materials such as polymers and sheets [14,15].

Despite the process shown in Figure 2 not being capable of attaching sheets to tube ends, there are three main guidelines that can be used in the design of the process to be presented in this paper: (i) the necessity of allowing the tube material to flow inwardly to produce a form-fit joint, (ii) the requirement of squeezing the sheets with deformation zone parameters within the range of Δ = 2–3 to obtain a combination of inhomogeneous and homogeneous plastic material flow and (iii) the applicability to sheets and tubes made from dissimilar materials.

The first two above-mentioned guidelines were recently used by the authors to develop a new joining by forming process (hereafter designated as ‘joining of sheets to tube ends by flaring and sheet squeezing’ or simply ‘joining by tube flaring and sheet squeezing’), that can attach metal sheets to metal tube ends without seal welding and machining of grooves in the holes of the sheet to obtain form-fit based joints [16].

**Figure 3:**
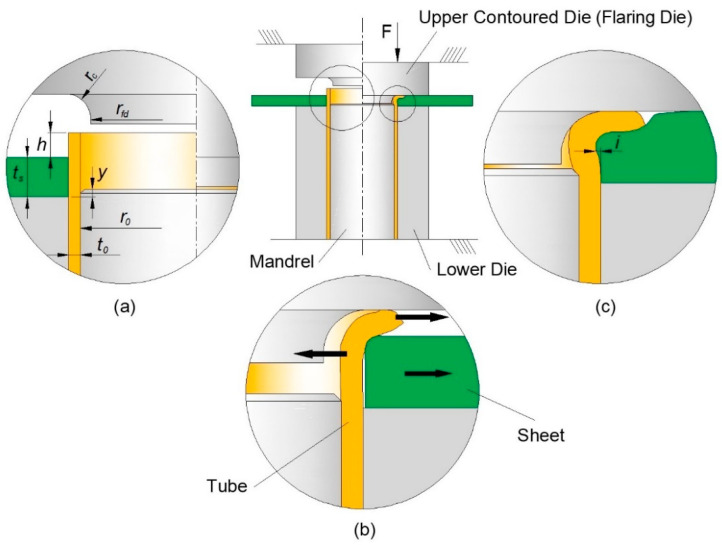
The new joining by forming process to perform single-stroke attachment of sheets to tube ends: (**a**) At the initial stage; (**b**) Flaring of the free tube end with the divided material flow; (**c**) Squeezing (indentation) of the tube flange against the sheet surface.

The new process is shown in Figure 3 and the aim and objective to be addressed in this paper is to extend its applicability domain to the attachment of sheets to tube ends made from polymers and metals. In particular, the paper seeks to analyze the main differences in the overall process deformation mechanics when the new process is applied to dissimilar materials with significant differences in strength. For this purpose, the work is focused on the connection of polymer sheets to metal tube ends, at ambient temperature.

The process will be comprehensively explained after this introduction by explaining the tool concept, identifying the major process parameters, and describing the methods and procedures utilized in the experimental and numerical simulation work. The working principle, typical modes of deformation, process variants with and without the use of mandrels and the required forming and destructive forces will be analyzed in the last section of the paper before listing the main conclusions and future work prospects.

## 2. Materials and Methods

### 2.1. Mechanical Characterization of the Materials

**Figure 4:**
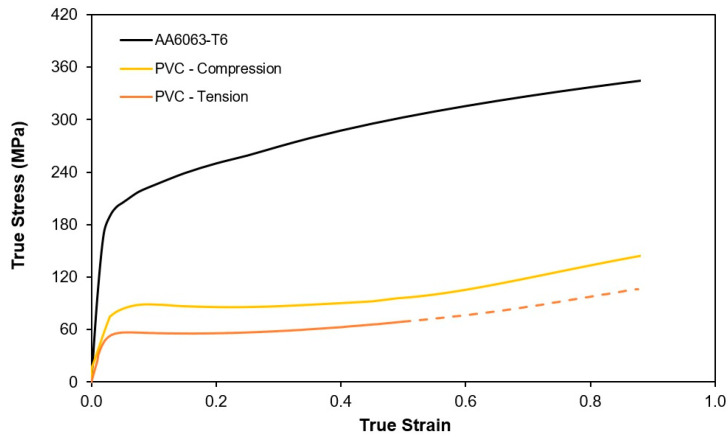
Flow curves of the PVC sheets and of the Aluminum AA6063-T6 tubes at ambient temperature.

The investigation on the extension of joining by tube flaring and sheet squeezing (Figure 3) to attachments made from dissimilar materials encompassed the use of Polyvinylchloride (PVC) sheets with 5 mm thickness (PolyLanema, Ovar, Portugal) and Aluminum AA6063-T6 tubes (Lanema, Ovar, Portugal) with 14.5 mm inner radius ($r_{o}$) and 1.5 mm wall thickness ($t_{o}$). The mechanical characterization of the materials at ambient temperature involved the combination of tensile and stack compression tests to obtain the stress responses up to values of true strain approximately equal to 0.8. The tensile tests were performed in accordance with the ASTM standards E8/E8M [17] and D638-14 [18] and the stack compression tests followed the general procedures described in [19]. The tests were carried out on a hydraulic testing machine (Instron SATEC 1200 kN, Norwood, MA, USA) with a crosshead velocity equal to 10 mm/min. and the experimental data utilized for plotting the flow curves shown in Figure 4 was retrieved from a previous mechanical characterization of the same materials performed by the authors [14].

The difference between the tensile and compressive behavior of the PVC sheets is due to the strength-differential effect that is typical of the cold plastic deformation of thermoplastics (such as PVC) at ambient temperature.

### 2.2. Fabrication of the Sheet-to-Tube End Attachments

The fabrication of the sheet-to-tube end attachments was carried out in the laboratory tool that is schematically illustrated in Figure 3. The tool was designed and constructed by the authors and was installed in the hydraulic testing machine that had been previously used in the mechanical characterization of the materials. The fabrication involved two different forming stages that were carried out sequentially in a single press stroke to obtain sheet-to-tube end attachment samples that are representative of the joining process:

(a) Firstly, the free tube end was flared by compression with a contoured die in such a way that divided material flow permitted the tube to move both inward and outward to create an appropriate geometry for subsequent mechanical interlocking (Figure 3b).
(b) Secondly, once the free tube end started to be squeezed (indented) against the sheet surface to produce a flange, material inside the sheet thickness started flowing inward and outward to create a mechanical interlocking (form-fit joint) and to allow the tube flange to plunge through the sheet to avoid material protrusions above the sheet surfaces at the end of the joining process (Figure 3c).

The experimental workplan utilized in the fabrication of the sheet-to-tube end attachments is summarized in Table 2 and involved variations in three main process parameters (Figure 3a): (i) the curvature radius $r_{c}$ of the contoured die (flaring die), (ii) the free tube height h above the sheet surface and (iii) the use of an internal mandrel.

Other process variables such as the inner radius $r_{fd}$ of the flaring die, and the vertical distance y of the upper mandrel end to the sheet surface took values from the previous work on the attachment of metal sheets to metal tube ends [16]. The geometry of the sheets and tubes was also kept constant to reduce the total number of variables.

At least three samples were prepared for each combination of parameters. After fabrication, selected samples were halved lengthwise to disclose the cross-sectional joints and to measure the interlocking distance i (Figure 3c) between the sheets and the tube ends.

### 2.3. Destructive Pull-Out Tests

**Figure 5:**
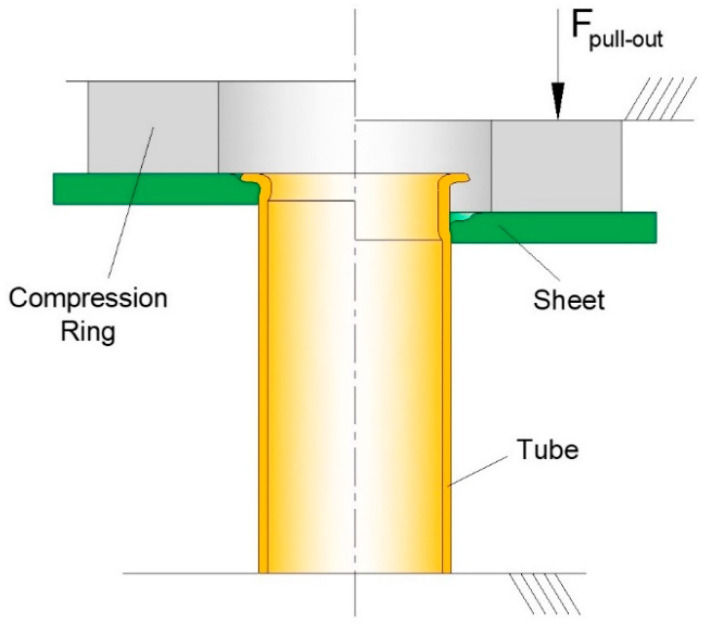
Schematic representation of a destructive pull-out test.

The performance of the attachments between the PVC sheets and the Aluminum tube ends were assessed by means of destructive pull-out tests. The tests consisted of detaching the tubes from the sheet holes by pushing the sheets with a flat compression ring, as it is schematically shown in Figure 5.

The experiments were carried out on the same hydraulic testing machine where mechanical characterization and joining by forming experiments were performed and using an identical crosshead velocity of 10 mm/min. The evolution of the force with displacement was registered for subsequent analysis.

### 2.4. Finite Element Modeling

The numerical simulation of the new joining by tube flaring and sheet squeezing process was carried out with the finite element computer program i-form [20]. The program was developed by the authors and is based on the finite element flow formulation, which is built upon the weak form of the quasi-static force equilibrium Equation (1),

(1) $$\int_{V}\sigma_{ij}\delta D_{ij}dV-\int_{S_{t}}t_{i}\delta u_{i}dS\;=\;0$$

In the Equation (1) $\sigma_{ij}$ is the Cauchy stress tensor, $D_{ij}$ is the rate of deformation tensor, $t_{i}$ denotes the tractions applied on the boundary $S_{t}$ with a normal with a vector of direction cosines given by $n_{j}$, and $\delta u_{i}$ is an arbitrary variation in the velocity because the flow formulation is written in terms of velocities.

Decomposition of the Cauchy stress tensor $\sigma_{ij}$ into a deviatoric tensor $\sigma_{ij}^{\prime }$ related to shape change and a hydrostatic tensor $\sigma_{m}\;=\delta_{ij}\sigma_{kk}/3$ related to volume change, in which $\delta_{ij}$ denotes the Kronecker delta, allows rewriting the weak form of the quasi-static force equilibrium Equation (2) as follows,

(2) $$\begin{array}{c} \int_{V}\sigma_{ij}^{\prime }\delta D_{ij}dV+\int_{V}\sigma_{m}\delta D_{v}dV-\int_{S_{t}}t_{i}\delta u_{i}dS\;=\;0\\ \Rightarrow \;\int_{V}\bar{\sigma }\delta \dot{\bar{\varepsilon }}dV+\int_{V}\sigma_{m}\delta {\dot{\varepsilon }}_{v}dV-\int_{S_{t}}t_{i}\delta u_{i}dS\;=\;0 \end{array}$$

In the Equation (2) ${\dot{\varepsilon }}_{v}\;=\;D_{v}\;=\;\delta_{ij}D_{ij}$ is the volumetric rate of deformation and $\sigma_{ij}^{\prime }\delta D_{ij}\;=\;\bar{\sigma }\delta \dot{\bar{\varepsilon }}$ is the increment of plastic power per unit of volume. The symbols $\bar{\sigma }$ and $\dot{\bar{\varepsilon }}$ denote the effective stress and effective strain rate according to the von Mises yield plasticity criterion in case of the Aluminum tubes and according to the Caddell et al. [21,22] plasticity criterion in case of the PVC sheets. The latter explicitly accounts for the strength-differential effect resulting from the differences between the flow stress of PVC in tension $\sigma_{T}$ and compression $\sigma_{C}$ (Figure 4) as follows,

(3) $${\bar{\sigma }}^{2}=\sigma_{C}\times \sigma_{T}-\left(\sigma_{C}-\sigma_{T}\right)\sigma_{kk}\;=\;0\;\;\;\;\;\;\;\;\;\sigma_{kk}\;=\;\delta_{ij}\sigma_{ij}$$

The computational approach to handle the second integral term in (2) is by relaxation of the incompressibility condition of the velocity field $\sigma_{m}\;=\;K{\dot{\varepsilon }}_{v}$, where K is a large positive number known as the ‘penalty’.

The extension of (2) to include friction and contact between different objects can be written as,

(4) $$\int_{V}\bar{\sigma }\delta \dot{\bar{\varepsilon }}dV+\int_{V}\sigma_{m}\delta {\dot{\varepsilon }}_{v}dV-\int_{S_{t}}t_{i}\delta u_{i}dS+\int_{S_{f}}\left(\int_{0}^{\left|u_{r}\right|}\tau_{f}\delta u_{r}\right)dS+K_{1}\overset{N_{c}}{\underset{c\;=\;1}{\sum }}g_{n}^{c}\delta g_{n}^{c}+K_{2}\overset{N_{c}}{\underset{c\;=\;1}{\sum }}g_{t}^{c}\delta g_{t}^{c}\;=\;0$$

where the symbols, $\tau_{f}$ and $u_{r}$ denote the friction shear stress and the relative sliding velocity on the contact interfaces $S_{f}$ between deformable and rigid bodies. The fifth and sixth terms in (4) account for the interaction between deformable bodies by means of a two-pass contact search algorithm in which the $N_{c}$ contact pairs are automatically extracted from the faces of the finite elements utilized in the discretization. The symbols $g_{n}^{c}$ and $g_{t}^{c}$ stand for the normal and tangential gap velocities in the contact pairs, which are penalized by large numbers $K_{1}$ and $K_{2}$ to avoid penetration. Details are given in Nielsen et al. [20].

**Figure 6:**
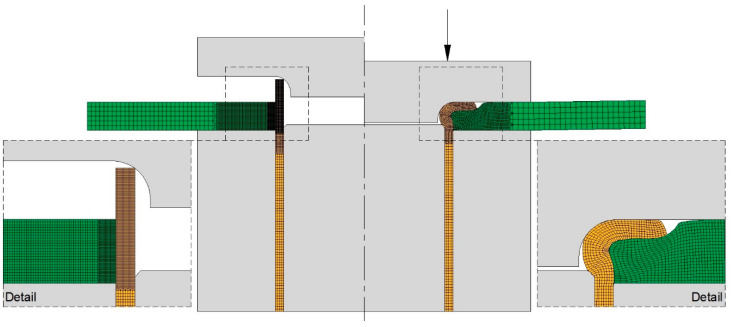
Finite element simulation details of the meshes in the beginning and at the end of the joining by forming process ($r_{c}=\;3$ mm and h = 6 mm).

The above-described procedure allowed modelling the sheet-to-tube end attachments as rotational symmetric objects with their cross-sections discretized by means of 1500 quadrilateral elements (Figure 6). The dies and mandrels were modelled as rigid objects and discretized by means of linear contact-friction elements.

A friction factor m equal to 0.1 was applied on the interfaces between the deformable and rigid objects after checking the finite element predicted joining forces that best matched the experimental measurements.

The central processing unit (CPU) time for a typical analysis using a convergence criterion for the velocity field and residual force equal to 10^−3^ was approximately 1.5 min. on a computer equipped with an Intel i7-5930K CPU processor (Santa Clara, CA, USA).

## 3. Results and Discussion

### 3.1. Modes of Deformation

**Figure 7:**
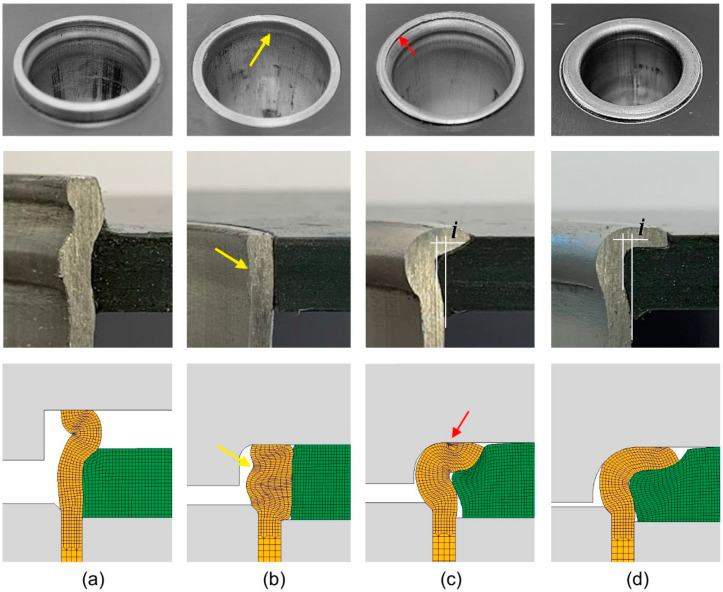
Experimental and finite element computed cross-sections of the typical modes of deformation that are observed in joining by tube flaring and sheet squeezing of Aluminum AA 6063-T6 tubes to PVC sheets: (**a**) Mode 0 ($r_{c}\;=\;0$ mm, h = 6 mm); (**b**) Mode 0 ($r_{c}\;=\;1$ mm, h = 4 mm); (**c**) Mode 1 ($r_{c}\;=\;2$ mm, h = 4 mm); (**d**) Mode 2 ($r_{c}\;=\;3$ mm, h = 4 mm).

Figure 7 discloses the three modes of deformation that were observed by varying the process parameters related to the curvature radius $r_{c}$ of the flaring die and the free tube height h. The results included in Figure 7a,b show two test cases in which plastic instability prevailed over divided flow. This happens when the free tube height h is very slender or when the curvature radius $r_{c}$ of the flaring die is very small. The phenomenon is more visible in Figure 7a because the initial free tube height h is higher than in Figure 7b. However, signs of plastic instability are still visible in Figure 7b by referring to the yellow arrows. Both Figure 7a,b is associated with combinations of process parameters that are unsuitable for producing sheet-to-tube end attachments. The associated plastic mode of deformation is hereafter named ‘mode 0’.

By increasing the curvature radius $r_{c}$ of the flaring die from 1 to 2 mm (Figure 7c), plastic instability will stop being the dominant mode of deformation and the tube material will start moving both inward and outward as the tube curls around the flaring die. Inward flow is necessary to create a form-fit joint, but as seen in the figure, it is energetically more favorable for the free tube height to increase its thickness than to create a mechanical interlocking between the sheet and the tube end.

As a consequence, and despite obtaining an experimental interference distance i= 0.7 mm, in fair agreement with the finite element predicted value i=0.4 mm, it is not recommended to use this mode of deformation (named as ‘mode 1’) for the attachment of sheets to tube ends. The small material overlap that develops on the tube flange (refer to the red arrows in Figure 7c) further contributes for not using this mode of deformation in sheet-to-tube end attachments.

A further increase in the curvature radius of the flaring die to a value $r_{c}\;=\;3$ mm allows obtaining the required sound form-fit attachment (Figure 7d). The experimental interference distance i = 0.8 mm, between the PVC sheet and the Aluminum tube, is larger and in fair agreement with the finite element predicted value i = 0.5 mm. This mode of deformation will be hereafter referred to as ‘mode 2’ and is recommended for the attachment of sheets to tube ends.

### 3.2. Joining with or without Inner Mandrel

**Figure 8:**
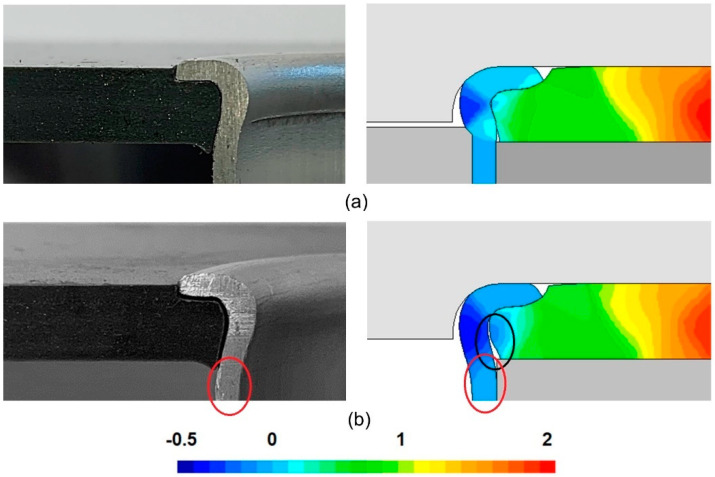
Experimental and finite element computed cross-sections of the joining by tube flaring and sheet squeezing of Aluminum AA 6063-T6 tubes to PVC sheets: (**a**) With an inner mandrel ($r_{c}\;=\;3$ mm, h = 4 mm); and (**b**) Without an inner mandrel ($r_{c}\;=\;3$ mm, h = 4 mm). The shaded finite element contour corresponds to the normalized radial velocity $v_{r}/v_{0}$.

The utilization of an inner mandrel helps creating form-fit joints with good mechanical interlocking (refer to Figure 7d) because: (i) plastic deformation is localized in the free tube height (Figure 8a) instead of propagating below the lower sheet surface (refer to the red ellipses in Figure 8b) and (ii) no significant gaps are formed along the contact interface between the tube and sheet hole (in contrast to what is seen in the black ellipse of Figure 8b).

The differences associated to the above-mentioned ellipses of Figure 8b are due to divided material flow with larger inward velocities, as shown in the normalized radial velocity $v_{r}/v_{0}$, where $v_{0}$ is the vertical velocity of the flaring die. Even so, it is possible to obtain a sheet-to-tube end attachment with good resistance to detachment (as will be confirmed later in the presentation), if the joining process is carried out without an inner mandrel. This opens the possibility of applying the new joining process in applications in which the access from the opposite tube end is difficult or impossible.

It should however be noted that the clearance between the sheets and the tubes in the photographs (namely in Figure 8b) is slightly exaggerated due to the elastic recovery of the polymer after halving the sheet and tube lengthwise to disclose the cross-sectional joint. Elastic recovery after halving is also responsible for the polymer surfaces not being coplanar with the tube flanges, as they appear in finite elements, and for the differences found between the experimental and finite element predicted interference distances of Section 3.1.

### 3.3. Joining and Destructive Forces

**Figure 9:**
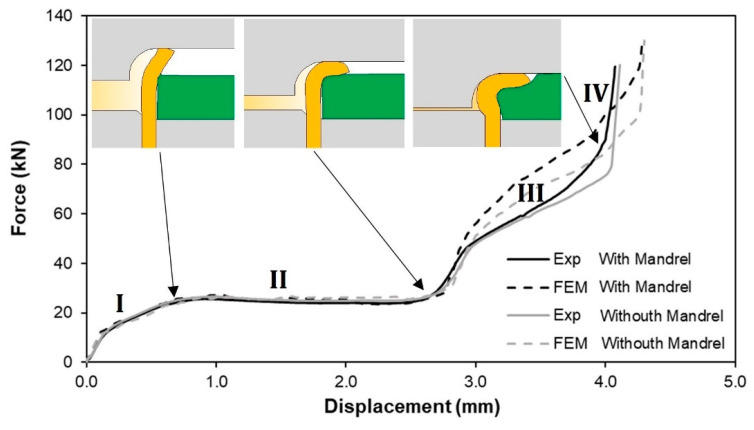
Experimental and finite element predicted evolution of the joining force vs. displacement with and without inner mandrel ($r_{c}\;=\;3$ mm, h = 4 mm).

Figure 9 shows the force vs. displacement evolutions for a typical deformation mode 2 of the sheet-to-tube end attachments obtained with and without an inner mandrel. The evolutions are characterized by four distinct regions:

(a) A first region (labelled as ‘I’) in which the force increases rapidly with the displacement due to the contact and beginning of the deformation of the tube end with the curvature radius $r_{c}$ of the flaring die,
(b) A second region (II) where the force is practically constant that corresponds to the curvature of the free tube height along with the flaring die,
(c) A third region (III) in which the tube flange that was in the meantime formed is progressively squeezed against the sheet surface to produce inner material flow within the sheet thickness and create a mechanical interlocking,
(d) A fourth and final region (IV) with a very sharp increase in force because of the flaring die starting to compress the sheet surface adjacent to the tube flange.

Regions I and II correspond to the ‘tube flaring’ stage, whereas regions III and IV are related to the ‘sheet squeezing’ stage of the joining process. The transition from ‘tube flaring’ into ‘sheet squeezing’ occurs progressively within the die stroke, as seen by the finite element details of the cross-sections that are included in the figure.

The influence of the inner mandrel on the evolution of the force with displacement is almost negligible. Major differences are found in region III (Figure 9) and correspond to a slight delay in the overall force response when the joining process is performed without a mandrel. These differences are attributed to a larger inward material flow of the free tube height during tube flaring without a mandrel (refer to Figure 8b), which leads to a smaller length of the tube flange that is squeezed against the sheet surface. Still, typical joining forces for both solutions (with and without a mandrel) are above 100 kN.

The agreement between the experimental and the finite element predicted evolutions of the force vs. displacement is good with major differences found in regions III and IV. These differences are related to variations in the submillimetre-range of displacements (below 0.2 mm) and are attributed to minor discrepancies between the real and the nominal dimensions of the tubes and sheets. In fact, very small variations of the inner radius, wall thickness and free height of the tubes as well as of the thickness and inner hole radius of the sheets may cause significant changes in the force vs. displacement response. However, despite the differences observed in regions III and IV, the overall trend and maximum values are similar.

**Figure 10:**
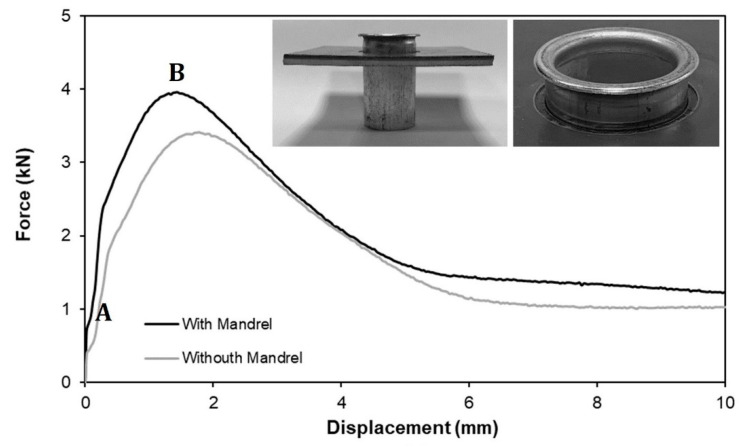
Experimental evolution of the pull-out destructive force vs. displacement with and without inner mandrel ($r_{c}\;=\;3$ mm, h = 4 mm).

Figure 10 shows the evolution of the destructive pull-out forces with displacement for a typical mode 2 sheet-to-tube end attachment obtained with and without an inner mandrel. As seen, the evolution is characterized by two distinct points:

(a) A kink labelled as ‘A’ that corresponds to the first irreversible relative motion between the sheet and the tube.
(b) A peak value labelled as ‘B’ where the maximum force corresponds to collapse by the detachment of the sheet from the tube.

Because the kink (‘A’) does not compromise the overall safety of the joint, the performance of the pull-out tests is analysed at the peak values ‘B’. It is worth noting that the photographs included in Figure 10 correspond to displacement values significantly beyond ‘B’.

Under these circumstances, the results shown in Figure 10 allow concluding that the utilization of a mandrel ensures cross-section joints with better pull-out resistance and peak values of approximately 4 kN. However, the peak value of 3.4 kN that was obtained for the pull-out resistance of the joints fabricated without a mandrel, also discloses the possibility of using this alternative procedure in applications where access from the opposite tube end is difficult or impossible.

## 4. Conclusions

Joining by flaring and sheet squeezing was successfully extended to the attachment of sheet to tube ends made from dissimilar materials (polymers and metals). The process is carried out in a single operation and the attachment is ensured by means of a form-fit (mechanical interlocking) based mechanism built upon divided material flow in both the tube and sheet materials. Divided material flow requires the inner radius of the flaring die to be smaller than the inner radius of the tube to allow the development of inward and outward radial velocities but also requires the curvature radius of the flaring die and the free tube height to be chosen to avoid failure by plastic instability or by thickening and overlapping (folding) of the free tube end.

The new joining process can be carried out with and without the utilization of an inner mandrel, although better pull-out destructive forces (4 kN) are achieved in case of the joints being fabricated with the use of an inner mandrel. Still, the performance of the joints fabricated without a mandrel (3.4 kN) is good enough to be used as an alternative in applications where access from the opposite tube end is difficult or even impossible.

The major limitations of the process are related to its application in tubes of very large wall thickness or in tubes made from low ductile materials, like in the commonly used joining by forming processes. Stringent requirements of leak tightness may also require the use of additional sealing procedures. Future developments will consider the application of the proposed process to a wider range of polymers and metals to gain a better insight of its limitations and potential industrial applications.

## Figures and Tables

**Table 1:** Main characteristics and features of the processes for attaching metal sheets to metal tube ends.

	Joining by Forming		Welding
Type of Joint	Force-Fit	Combined Force-Fit and Form-Fit	Fusion-Based
**Processes**	Hydraulic, mechanical, or explosive expansion of tubes (combined with seal welding)	Hydraulic, mechanical, or explosive expansion of tubes	Strength welding by means of gas tungsten arc welding (GTAW)
**Mechanism**	Friction due to interfacial pressure (combined with seal welding)	Friction due to interfacial pressure combined with mechanical interlocking	Melting with addition of filler materials
**Preparation**	Cleaning	Cleaning and machining of grooves in the sheet hole	Cleaning and edge preparation
**Joining temperature**	Ambient temperature (combined with melting temperature during seal welding)	Ambient temperature	Melting temperature
**Heat-affected zones**	No/Yes (distortion and changes in microstructure due to thermal cycles of seal welding)	No	Yes (distortion and changes in microstructure due to thermal cycles)
**Materials**	Dissimilar metals/Similar metals (when combined with seal welding)	Dissimilar metals	Similar metals
**Relative Productivity**	High/Medium (use of clamps and jigs during seal welding)	Medium	Low (use of clamps and jigs plus the need of inspection)
**Environmental friendliness**	High/Medium (toxic fumes, smoke, dust particles, difficult replacement, or detachment of tubes)	Medium (difficult replacement or detachment of tubes)	Low (toxic fumes, smoke, dust particles, difficult replacement, or detachment of tubes)

**Table 2:** Experimental work plan for the joining by tube flaring and sheet squeezing. Notation in accordance with Figure 3.

Tube (Aluminum AA 6063-T6)			Sheet (PVC)	Flaring Die		Mandrel	
$r_{o}$ (mm)	$t_{o}$ (mm)	h (mm)	$t_{s}$ (mm)	$r_{fd}$ (mm)	$r_{c}$ (mm)	Utilization	y (mm)
14.5	1.5	2–6	5	13.25	0–3	Yes/No	1

## Data Availability

Data sharing is not applicable to this article.
